# The Association of Having a Late Dinner or Bedtime Snack and Skipping Breakfast with Overweight in Japanese Women

**DOI:** 10.1155/2019/2439571

**Published:** 2019-03-03

**Authors:** Chika Okada, Hironori Imano, Isao Muraki, Keiko Yamada, Hiroyasu Iso

**Affiliations:** ^1^Department of Nutritional Epidemiology and Shokuiku, National Institutes of Biomedical Innovation, Health and Nutrition, Tokyo, Japan; ^2^Public Health, Department of Social Medicine, Osaka University Graduate School of Medicine, Osaka, Japan; ^3^Department of Public Health and Medical Affairs, Osaka Prefectural Government, Osaka, Japan

## Abstract

**Background:**

We aimed to assess the association of habitually eating in the late evening and skipping breakfast with the prevalence of overweight/obesity.

**Methods:**

A total of 19,687 Japanese women, aged 40–74 years, were asked about their height, weight, and habitual eating behaviors such as having a late dinner and a bedtime snack and skipping breakfast, using a self-administered questionnaire. We defined overweight/obesity as body mass index greater than or equal to 25 kg/m^2^.

**Results:**

Among the participants, 11% regularly had a late dinner, 22% had bedtime snacks, and 8% skipped breakfast. After adjusting for age, exercise, smoking, sleep duration, and employment, the multivariable-adjusted odds ratios (ORs) and 95% confidence intervals (CIs) of skipping breakfast were 2.47 (2.18–2.81) for having a late dinner and 1.71 (1.53–1.91) for having a bedtime snack. These eating behaviors were associated with an increased risk of overweight/obesity: the multivariable-adjusted ORs (95% CIs) of obesity/overweight were 1.43 (1.27–1.62) for having a late dinner, 1.47 (1.34–1.62) for having a bedtime snack, and 1.23 (1.06–1.42) for skipping breakfast.

**Conclusions:**

Japanese women who consumed late dinners or bedtime snacks were more likely to skip breakfast. Having a late dinner or bedtime snack was associated with a higher probability of overweight/obesity.

## 1. Introduction

Obesity is recognized as a worldwide health issue, and its age-standardized prevalence has increased from 6.4% to 14.9% in women and from 3.2% in 1975 to 10.8% in 2014 in men [1]. If obesity were to continue increasing at the same rate, its prevalence will reach 21% in women and 18% in men by 2025 [1]. Recently, the Global Burden of Disease 2015 Obesity Collaborators reported that high body mass index (BMI) contributed to 4.0 million deaths and 120 million disability-adjusted life-years in 2015 [2]. Elderly people with BMI 30 kg/m^2^ or higher, or those in the highest quintile of percent body fat, also had a significantly higher risk of functional limitation [3, 4]. This is noteworthy because, even in Japan where residents generally live long, healthy lives, healthy life expectancy is 9 years shorter than the actual lifespan in men and 11 years shorter than the actual lifespan in women [5]. Therefore, to extend healthy life expectancy, preventing overweight/obesity is an urgent public health concern in an aging world.

In previous cross-sectional [6–9] and cohort studies [10–12], skipping breakfast was associated with overweight/obesity and weight gain. Although skipping breakfast is a widespread behavior that is increasing in prevalence, possibly leading to overweight/obesity [13–15], there is limited evidence to examine the influence of other meals on skipping breakfast [16]. Our hypotheses are that individuals who eat something at night are more likely to subsequently skip breakfast and that late-night eating is associated with overweight/obesity, when analyzing dietary behaviors both in the night and in the morning. Eating at night has also been associated with a risk of obesity in a cohort study, but that study did not consider breakfast [17]. The aim of the present study was to test our hypotheses among middle-aged Japanese subjects.

## 2. Methods

### 2.1. Study Population

We used anonymized secondary data from a questionnaire-based health survey of dependents of employees insured by the Osaka branch of the Japan Health Insurance Association. According to the Health Insurance Act in Japan, dependents must be primarily receiving financial support from an employed family member, with no regular work and low annual income. In 2015, participants, 40–74 years of age, were mailed a self-administered questionnaire on their health and health-related behaviors from the Osaka prefecture office, and a total of 23,122 individuals responded to the questionnaire. We excluded participants with missing information on age and sex (*n* = 347), those who were missing data on skipping breakfast, having bedtime snacks, and having late dinner (*n* = 354), those who did not report body height and weight (*n* = 2,200), and male participants due to their small number (*n* = 534). In total, we included 19,687 middle-aged Japanese women in the current analysis.

Informed consent was not explicitly obtained, but participants were considered to have agreed to participate in the survey if they had filled out the questionnaire. This study has been exempted from Institutional Review Board approval due to its secondary use of anonymous data.

### 2.2. Assessment of Eating Behaviors and Covariates

In the self-administered questionnaire, three eating behaviors (having a bedtime snack, having a late dinner, and skipping breakfast) during the last six months were assessed with the following questions: (1) “Do you have a snack after dinner (bedtime snack other than three regular meals) at least three times per week?” for having a bedtime snack, (2) “Do you have dinner within 2 h before bedtime at least three times per week?” for having a late dinner, and (3) “Do you skip breakfast at least three times per week?” for skipping breakfast.

We also asked participants about their age, body height and weight, exercise habits of ≥30 minutes/day and ≥2 days/week (yes or no/prohibited), smoking status (never, former or currently smoking), average daily sleep duration (<5, 5–6, 6–7, 7–8 or ≥8 hours), and employment status. Body mass index (BMI) was calculated as weight (kg) divided by height squared (m^2^), and overweight was defined as having a BMI of 25 kg/m^2^ or greater.

### 2.3. Statistical Analyses

The odds ratios (ORs) and 95% confidence intervals (CIs) of skipping breakfast and overweight were estimated by logistic regression models. Regarding the associations with both skipping breakfast and overweight, we adjusted for age, exercise habits, smoking status, sleep duration, and employment (model 1). For the association with overweight, we also mutually adjusted for both eating behaviors, i.e., having a late-night meal and skipping breakfast, in addition to adjustments for the covariates used in model 1, although such behaviors might be mediators of overweight. Having a late-night meal was defined as either having a bedtime snack or a late dinner because of the small number (4.6 %) of participants who reported both eating behaviors (model 2). Two-sided *p* values below 0.05 were considered statistically significant. All statistical analyses were performed using SAS version 9.4 (SAS Institute Inc.).

## 3. Results

As shown in Table 1, approximately one-third of the participants habitually exercised, and almost half of the participants had a job. Of the participants, 11% habitually had a late dinner, 22% habitually had bedtime snacks, and 8% habitually skipped breakfast.

Both eating behaviors of having a late dinner and having a bedtime snack were associated with a higher probability of skipping breakfast (Table 2). The age-adjusted ORs (95% CIs) of skipping breakfast were 2.80 (2.48–3.16) for having a late dinner and 1.75 (1.57–1.95) for having a bedtime snack, compared with those who did not. Even after adjusting for health-related behaviors and employment status, these associations were only slightly weakened and remained statistically significant: the multivariable-adjusted ORs of skipping breakfast were 2.47 (2.18–2.81) for having a late dinner and 1.71 (1.53–1.91) for having a bedtime snack.

Table 3 shows the association between each eating behavior (late dinner, nighttime meal, or skipping breakfast) and overweight/obesity. All three of these eating behaviors were significantly associated with higher prevalence of overweight after adjustment for health-related behaviors and employment status: the multivariable-adjusted ORs (95% CIs) of overweight were 1.46 (1.29–1.65) for having a late dinner, 1.48 (1.35–1.63) for having a bedtime snack, and 1.29 (1.12–1.49) for skipping breakfast. After further mutual adjustment for having late meals (late dinner and/or bedtime snack) and skipping breakfast, the associations for overweight were attenuated slightly while maintaining statistical significance: the multivariable ORs (95% CIs) were 1.43 (1.27–1.62) for having a late dinner, 1.47 (1.34–1.62) for having a bedtime snack, and 1.23 (1.06–1.42) for skipping breakfast. Furthermore, the combination of eating late in the evening (having a late dinner and/or bedtime snack) plus skipping breakfast was strongly associated with a higher prevalence of overweight/obesity: the multivariable OR (95% CI) was 1.63 (1.33–2.00) for participants exhibiting both eating behaviors, compared with those with neither behavior (Table 4).

## 4. Discussion

In this cross-sectional study of Japanese women, we found that the habitual eating behaviors of having dinner within 2 hours before bedtime and having a bedtime snack were associated with a higher probability of skipping breakfast, and they were also associated with a higher prevalence of overweight independently of skipping breakfast. One notable observation of our study is that, compared to skipping breakfast, eating a late dinner and having a bedtime snack were more strongly associated with higher prevalence of overweight.

Night eating was more frequent among individuals with obesity than among those without, and such individuals are more likely to engage in binge eating [18]. Furthermore, greater energy intake in the late evening has been associated with a higher risk of obesity [19, 20]. Healthy individuals [17] and women with obese [21] who engage in night eating gain more weight than those who do not. On the contrary, breakfast intake was associated with a lower risk of weight gain [19] and obesity [22] in prospective studies. Only one study simultaneously assessed the behaviors of eating late-night meals and skipping breakfast with obesity defined as BMI ≥30 kg/m^2^ [23], and to our knowledge, there are no studies that assessed these associations using a BMI cutoff point of 25 kg/m^2^ as defined by Japanese criteria of obesity.

Our study predictably indicated a significant association of nighttime eating behaviors, such as having a late dinner and having a bedtime snack, with skipping breakfast, and both eating behaviors at night and in the morning were independently associated with a higher risk of overweight. One of the mechanisms for this observation is related to the definition of energy balance as the relationship between energy intake and energy expenditure. Nighttime eaters may consume more calories per day than those who do not eat at night [17], and eating at night appears to have a lower energy expenditure than eating in the morning, as a result of lower diet-induced thermogenesis and a lower resting metabolic rate after meals [24, 25]. Another explanation might be circadian misalignment, where awaking and eating at night might result in a decreased plasma leptin concentrations and increased glucose levels [26, 27]. To prevent overweight/obesity, the timing of meals should be considered, e.g., avoiding late dinners and nighttime meals.

A major strength of our study is that it is a large population-based study of women in Japan. However, our study has some limitations. Firstly, we cannot conclude the causality of the current findings because it is a cross-sectional study. Secondly, we had no data on overall dietary intake, and so we cannot examine whether nighttime eating was associated with overweight independently of dietary factors such as energy intake, glycemic index, and glycemic load. Thirdly, height and weight were self-reported. Since self-reported height and weight are highly correlated with measured height and weight in the Japanese population [28], the misclassification of overweight may be limited. Next, we cannot deny the possibility of residual confounding factors, although we adjusted for some risk factors of obesity such as short sleep [29–32]. Lastly, the generalizability of the current findings may be limited to the Japanese population.

## 5. Conclusions

Both having a late dinner and having a bedtime snack were associated with the prevalence of skipping breakfast. These nighttime eating behaviors, as well as skipping breakfast, were independently associated with a higher prevalence of overweight. Our results suggest that modification of these eating behaviors may contribute to the prevention and control of overweight/obesity.

## Figures and Tables

**Table 1:** The characteristics of study participants.

	Proportion or median (interquartile range)
Number	19,687
Age, median	50 (43–60)
40–49 (%)	47.9
50–59 (%)	25.6
60–69 (%)	23.1
70–74 (%)	3.5
Body mass index (kg/m^2^)	21.2 (19.6–23.2)
Exercise habit (%)	31.5
Current smoker (%)	7.7
Sleep duration (%)	
<5.0 hours	8.7
5.0–5.9 hours	38.9
6.0–6.9 hours	36.8
7.0–7.9 hours	12.6
8.0–8.9 hours	2.4
≥9.0 hours	0.3
Employed (%)	50.2
Having late dinner (%)	11.3
Having bedtime snack (%)	22.2
Skipping breakfast (%)	8.4

**Table 2:** Odds ratios (ORs) and 95% confidence intervals (CIs) of skipping breakfast for having a late dinner and having a bedtime snack.

	Having late dinner		Having bedtime snack	
	No	Yes	No	Yes
No. of participants	17,456	2,231	15,323	4,364
No. of participants skipping breakfast	1,252	407	1,115	544
Age-adjusted OR	1.00	2.80 (2.48–3.16)	1.00	1.75 (1.57–1.95)
Multivariable OR	1.00	2.47 (2.18–2.81)	1.00	1.71 (1.53–1.91)

Multivariable OR was adjusted for age, exercise habit, smoking status, sleep duration, and employment status.

**Table 3:** Odds ratios (ORs) and 95% confidence intervals (CIs) of overweight/obesity for having a late dinner, having a bedtime snack, and skipping breakfast.

	Having late dinner	Having bedtime snack	Skipping breakfast
Overweight in participants without unhealthy behavior (%)	12.5	12.0	12.8
Overweight in participants with unhealthy behavior (%)	16.9	16.5	15.7
Age-adjusted OR (95% CI)	1.48 (1.32–1.67)	1.52 (1.38–1.67)	1.36 (1.18–1.57)
Multivariable OR (95% CI)^a^	1.46 (1.29–1.65)	1.48 (1.35–1.63)	1.29 (1.12–1.49)
Multivariable OR (95% CI)^b^	1.43 (1.27–1.62)	1.47 (1.34–1.62)	1.23 (1.06–1.42)

Overweight was defined as a body mass index of 25 kg/m^2^ or greater. Unhealthy behaviors included having a late dinner, having a bedtime snack, or skipping breakfast. ORs were calculated for having certain eating behaviors compared with those without the same eating behavior. ^a^Adjusted for age, exercise habit, smoking status, sleep duration, and employment status. ^b^Adjusted additionally and mutually for having a late-night meal (late dinner and/or bedtime snack) and skipping breakfast.

**Table 4:** Odds ratios (ORs) and 95% confidence intervals (CIs) of overweight/obesity according to combinations of having a late dinner or a bedtime snack and skipping breakfast.

	Having late dinner or bedtime snack/skipping breakfast			
	No/no	Yes/no	No/yes	Yes/yes
No. of participants	13,073	4,955	915	744
No. of overweight participants	1,522	778	132	128
Age-adjusted OR (95% CI)	1.00	1.47 (1.34–1.62)	1.37 (1.13–1.66)	1.76 (1.44–2.14)
Multivariable OR (95% CI)	1.00	1.45 (1.32–1.60)	1.32 (1.08–1.61)	1.63 (1.33–2.00)

Overweight was defined as a body mass index of 25 kg/m^2^ or greater. Multivariable OR was adjusted for age, exercise habit, smoking status, sleep duration, and employment status.

## Data Availability

The dataset used to support the findings of this study have not been made available because of the collaboration agreement on the survey.
